# Supplementary material for: Comprehensive Annotation and Functional Exploration of MicroRNAs in Lettuce
Source: Front Plant Sci. 2021 Dec 24;12:781836. doi: 10.3389/fpls.2021.781836 (PMC8739914; doi:10.3389/fpls.2021.781836)
Supplement: Supplementary file 3 [file Data_Sheet_9.ZIP › GSM2230751.plot/Lsa-miRN1_Lsat_1_v5_gn_4_3221.1_582_TPlot.pdf]

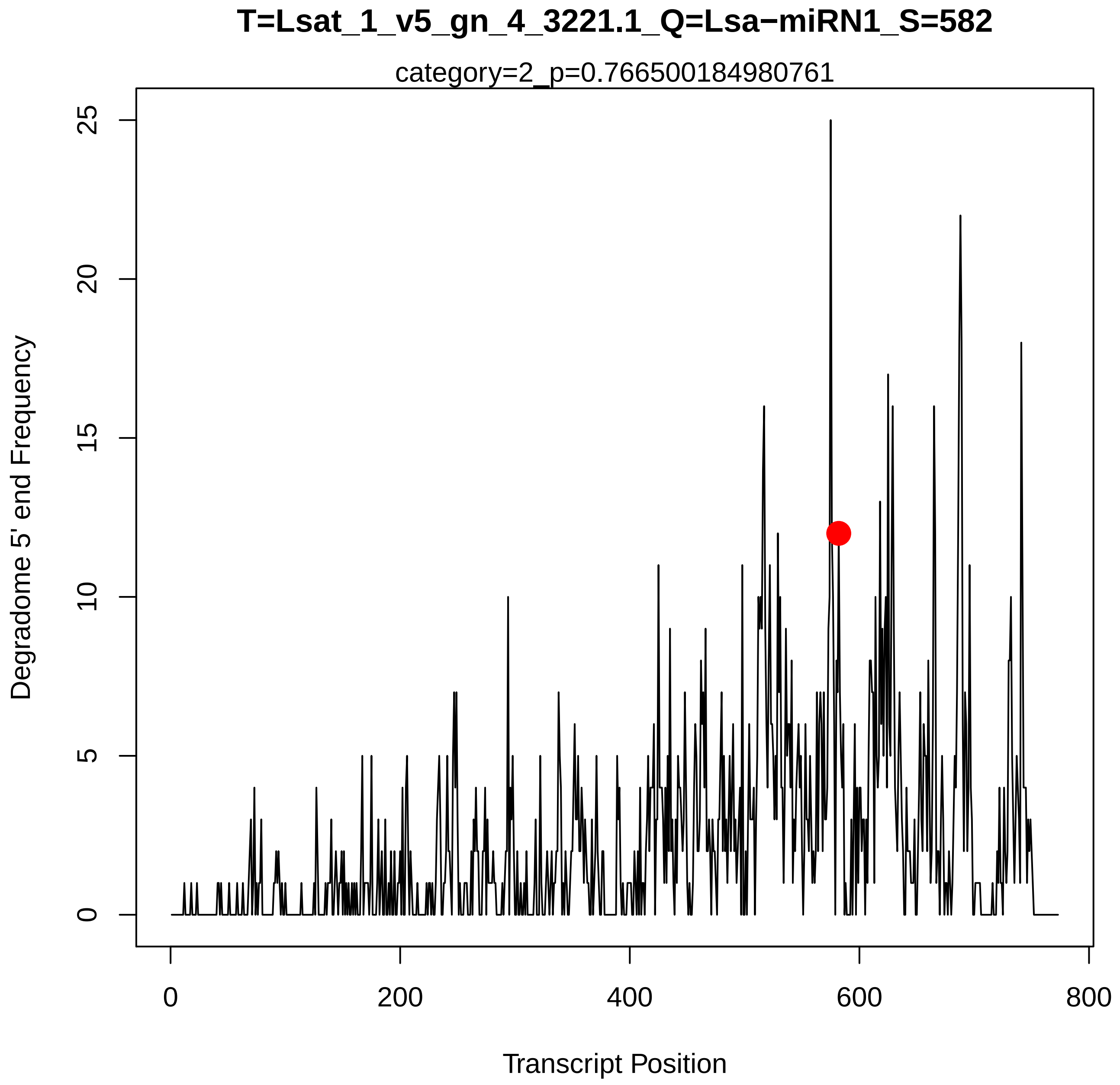
T=Lsat_1_v5_gn_4_3221.1_Q=Lsa−miRN1_S=582
category=2_p=0.766500184980761
Degradome 5' end Frequency
Transcript Position
0
5
10
15
20
25
0
200
400
600
800
